# Responses of canine periodontal ligament cells to bubaline blood derived platelet rich fibrin in vitro

**DOI:** 10.1038/s41598-021-90906-z

**Published:** 2021-06-01

**Authors:** Poranee Banyatworakul, Thanaphum Osathanon, Sujin Chumprasert, Prasit Pavasant, Nopadon Pirarat

**Affiliations:** 1 Department of Pathology, Faculty of Veterinary Science, Chulalongkorn University, Bangkok, 10330 Thailand; 2 Dental Stem Cell Biology Research Unit, Faculty of Dentistry, Chulalongkorn University, Bangkok, 10330 Thailand; 3 Department of Anatomy, Faculty of Dentistry, Chulalongkorn University, Bangkok, 10330 Thailand; 4 Oral Biology Research Center, Faculty of Dentistry, Chulalongkorn University, Bangkok, 10330 Thailand; 5 Wildlife Exotic and Aquatic Pathology-Research Unit, Department of Pathology, Faculty of Veterinary Science, Chulalongkorn University, Bangkok, 10330 Thailand

**Keywords:** Cell biology, Medical research

## Abstract

Platelet-rich fibrin (PRF) promotes wound healing by providing the release of growth factors. Here, the influence of Thai and Murrah bubaline blood derived PRF on canine periodontal ligament cells (cPDLs) was investigated. PRF was prepared from Thai and Murrah buffaloes with single centrifugation. Results demonstrated that Thai bubaline blood derived PRF exhibited fiber-mesh like morphology and contained more platelet entrapment than Murrah bubaline blood derived PRF. Both bubaline PRFs were able to degrade in vitro under condition with trypsin. Thai but not Murrah bubaline blood derived PRF promoted cPDLs proliferation in serum free and 2% serum culture conditions. Correspondingly, the significant upregulation of *KI67* mRNA expression was observed in those cells treated with Thai bubaline blood derived PRF. However, both Thai and Murrah bubaline blood derived PRF accelerated cell migration in an in vitro wound healing assay and facilitated cell spreading. Further, cPDLs cultured in osteogenic induction medium supplemented with Thai bubaline blood derived PRF exhibited the increased mineral deposition in vitro. Frozen Thai bubaline blood derived PRF also promoted cell proliferation, *KI67* mRNA expression, cell migration, and cell spreading in cPDLs. Taken these evidence together, bubaline blood derived PRF could provide potential benefits for canine periodontal tissue healing.

## Introduction

Periodontal ligament is a fibrous connective tissue locating between cementum and alveolar bone. It functions to support and to secure tooth in the alveolar bone socket. Besides, periodontal ligament contributes crucial roles in biological responses to stimuli and in maintaining homeostasis. Periodontal disease is one of the most common oral diseases in human and small animals. The destruction of the periodontal apparatus namely gingiva, cementum, periodontal ligament, and alveolar bone is occurred as a result of inflammatory process. Similar to human, periodontal disease is one of the common oral diseases in dogs, leading to the periodontal destruction. Over eighty percent of dogs older than 2 years-old exhibit signs of periodontal disease^1^. The effect of periodontal disease is not only localized in oral cavity, but also generalized contribute to several systemic conditions^2,3^. Inflammatory processes involved a disease’s progression by releasing of cytokines and chemokines. This process plays a crucial role in periodontal pathogenesis. Gold standard of periodontal treatment is to eliminate inflammatory process and to allow periodontal tissue regeneration. The conventional treatment, professional dental scaling and root planning are chosen to control the progression of the disease. Subsequently, several approaches have been utilized in order to promote periodontal tissue regeneration for example bone replacement grafts, guided tissue regeneration, and biologic mediators^4–8^. However, with limitation of current treatment outcomes, several attempts have been rigorously investigated to minimize inflammatory process, to stimulate periodontal tissue regeneration, and to accelerate wound healing.

Platelet-rich fibrin (PRF), a second generation of platelet derived agents, was developed to improve the preparation of the previous generation. PRF is considered as a healing biomaterial presented with clumping of platelets, leucocytes, growth factors and cytokines trapped in a fibrin meshwork. PRF releases many growth factors including platelet derived growth factor (*PDGF*), transforming growth factor ß1 (*TGFB1*), vascular endothelial growth factor (*VEGF*), insulin-like growth factor-1 (*IGF1*), fibroblast growth factor (*FGF*), and epidermal growth factor (*EGF*)^9^. These growth factor is gradually released during 7 days observation period^10^. These growth factors facilitate numerous wound healing processes, including chemotaxis, inflammation, proliferation of granulation tissue, reepithelization, extracellular matrix formation, and remodeling^11^. It has been reported that PRF reduced pain and inflammation on oral wound^12–14^.

In veterinary treatment, surgical dental extraction is the conservative treatment in dogs with advanced periodontitis. However, animals suffer from bleeding, trauma, infection, and a prolonged hospitalization in veterinary hospitals. Gingivectomy and apically repositioned flap are the alternative surgical techniques in dogs to maintain the affected tooth by reducing depth of the periodontal pocket. An adjuvant treatment with bioactive agents aiming to minimize inflammation and to promote periodontal wound healing is introduced in canine periodontal treatment. The preparation of PRF is an easy and cost-effective way in clinical use in human study. Our previous report demonstrated the use of autologous PRF in periodontal regenerative treatment in dogs^15^. However, a high-volume collection of autologous blood in small dog breeds has technical difficulty. In addition, all dental procedures in dogs must be performed under general anesthesia. Hence, an alternative source for PRF preparation is indeed required. The morphology and mechanical properties of PRF depend on the amount and architecture of fibrinogen and thrombin. Bubaline blood is proposed as an alternative fibrinogen source. It exhibits the highest fibrinogen compared with those from human, bovine, and ovine^16^. Fibrinogen interacts with β3 integrin on platelet membrane leading to platelet aggregation. Further, platelets are known source of growth factor and cytokine facilitating healing and regeneration. In addition, bubaline fibrinogen have been used in human clinical study for periodontal surgery^17^. Hence, development of bubaline PRF would be beneficial in term of readiness in clinical treatment and overcome the high-volume blood collection in small animals. To date, the efficacy of PRF from animal blood donation has not been fully reported. Therefore, bubaline fibrinogen might be a potential source of platelet concentrate evolution. As our ultimate aim is to use bubaline PRF in periodontal regeneration in dogs. Thus, the objective of this study was to assess the biological effects of bubaline PRF on canine periodontal ligament cells (cPDLs) regarding cell attachment, spreading, proliferation, migration and osteogenic differentiation.

## Results

### Gross morphology and Ultrastructure examination of Bubaline blood derived PRF

**Figure 1:**
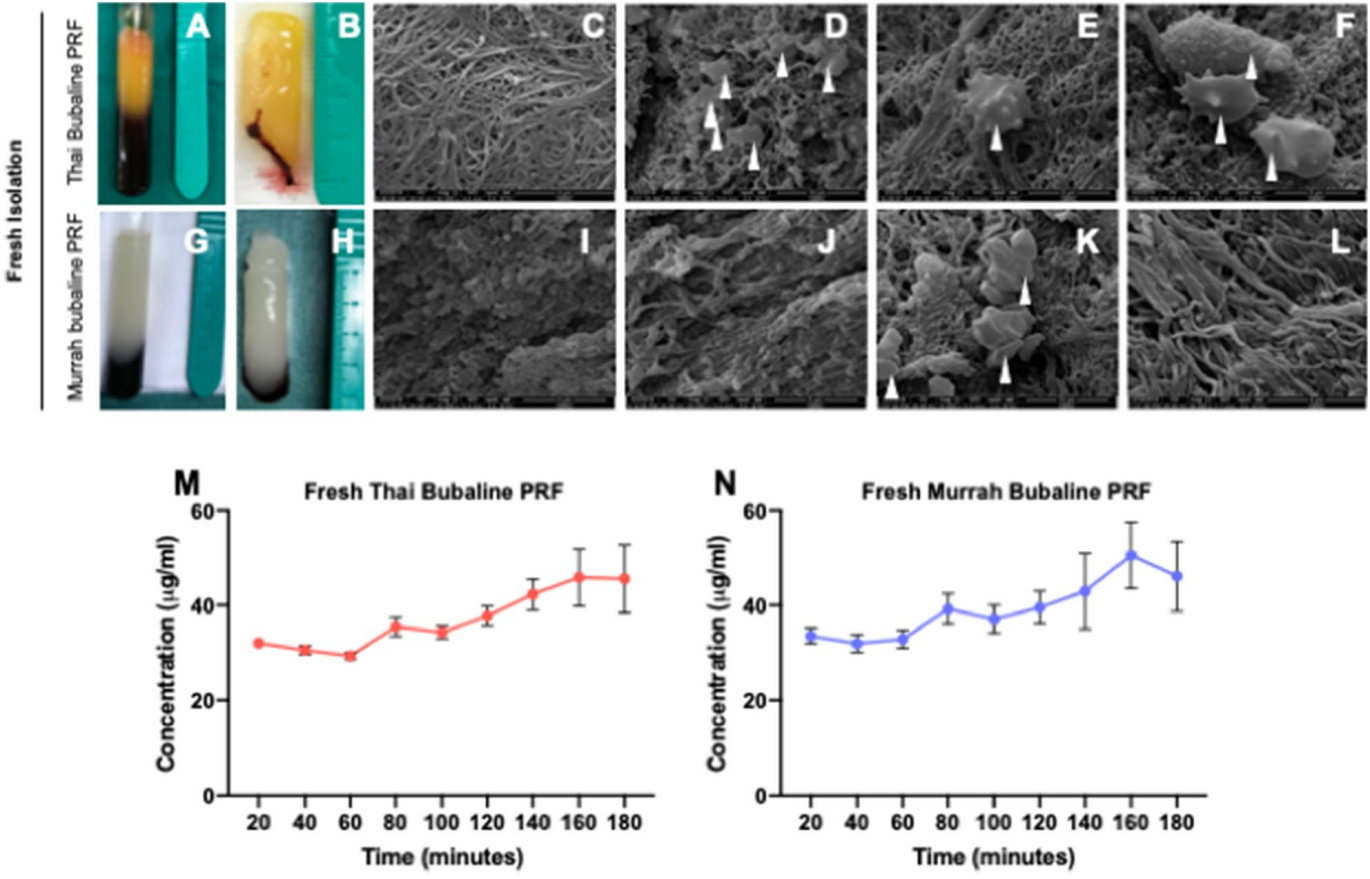
Gross and ultrastructure examination of platelet rich fibrin (PRF). Gross structure examination of PRF derived from Thai (**A** and **B**) bubaline blood exhibited yellowish colored membrane but Murrah bubaline blood derived PRF (**G** and **H**) demonstrated lighter color. Ultrastructure examination by scanning electron microscope revealed that PRF derived from Thai bubaline blood (**C**–**F**) had higher platelet entrapment than Murrah bubaline blood-derived PRF (**I**–**L**). Arrow heads indicated platelets and white blood cells. After PRFs were incubated with trypsin solution at 37 °C, protein concentration in supernatant was measured using BCA assay at different time points. Results (**M**–**N**) illustrated the degradation ability of those PRF membranes.

**Figure 2:**
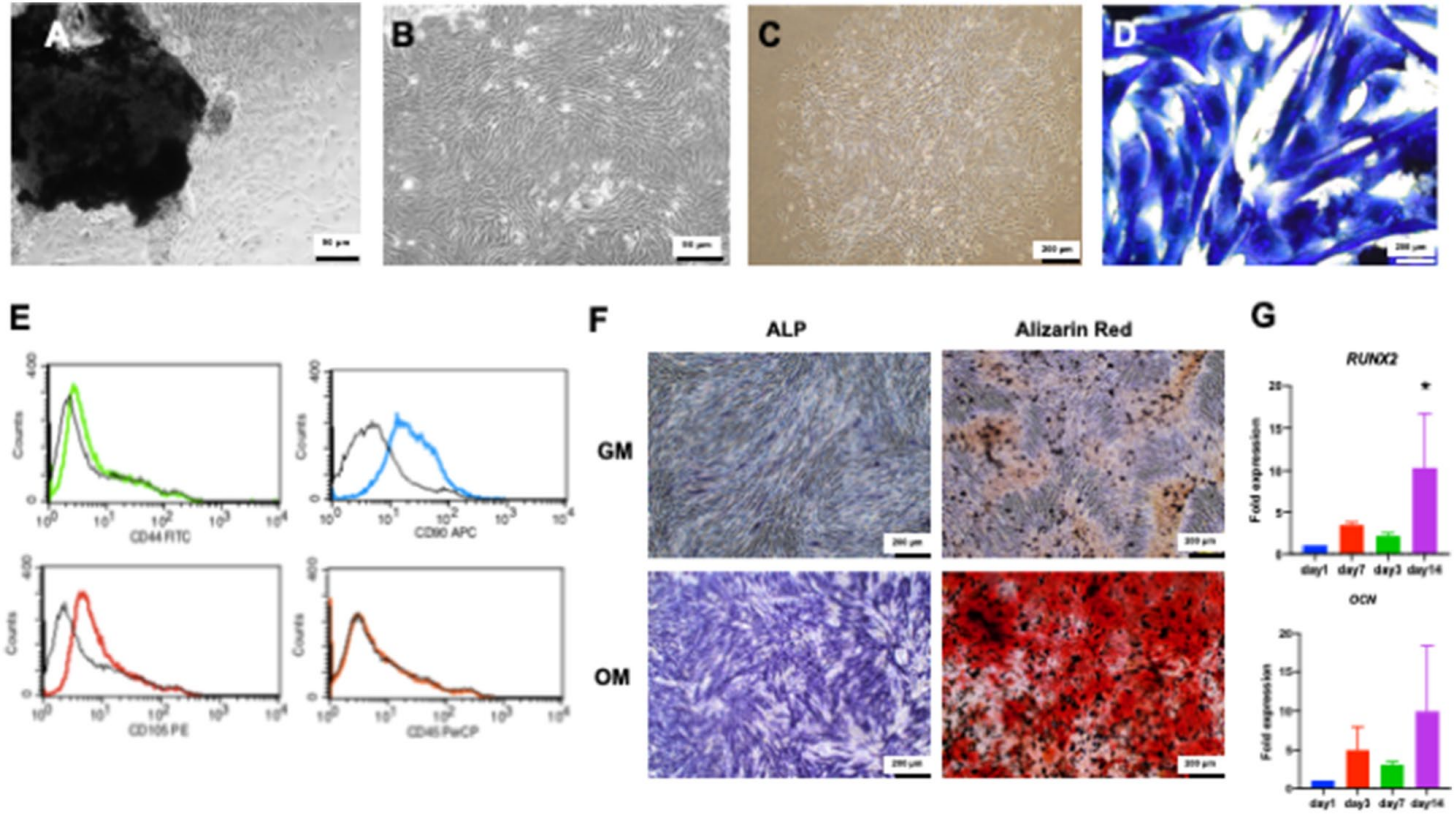
Characterization of canine periodontal ligament cells (cPDLs). cPDLs were isolated using cell explantation. Cell migration out from the tissue was observed at day 14 (**A**). These cells exhibited fibroblast morphology (**B**) and able to form colonies (**C**). Colonies were stained with Coomassie blue to present the homogeneous cell morphology (**D**). The isolated cells expressed CD44, CD90, and CD105 but not CD45 as evaluated by flow cytometry analysis (**E**). After maintaining cPDLs in osteogenic induction medium, ALP expression and mineral deposition was increased as determined by ALP enzymatic activity staining and alizarin red s staining, respectively (**F**). GM; growth medium, OM; osteogenic induction medium. The upregulation of osteogenic marker gene expression was investigated using real-time PCR (**G**). Asterisks indicated the statistically significant difference compared with day 1. (Kruskal–Wallis test with Dunn’s multiple comparisons test, *p* < 0.05).

Ten millimeters of whole blood was separated into three compartments after single centrifugation for 10 min at 3000 rpm. PRF was harvested from a yellowish color membrane of fibrin clot separating from acellular supernatant and red corpuscles in the bottom of test tube. PRF clots were easily handled when the acellular supernatant and red blood cells were removed. Ratio of PRF and red corpuscles is approximately 1:1 for Thai bubaline blood (Fig. 1A and B) and 2:1 for Murrah bubaline blood (Fig. 1G and H).

SEM analysis revealed dense fibrin rich matrix in both structures of Thai (Fig. 1C–F) and Murrah (Fig. 1I–L) bubaline PRF. A number of leucocytes and platelets trapped in three-dimensional fibrin networks were observed in both conditions. Thai bubaline PRF exhibited more fibrin mesh morphology and contained higher number of platelet entrapment than Murrah bubaline PRF. In addition, a higher number of white blood cells was noted in Thai bubaline PRF. From degradation study, both Thai and Murrah bubaline PRF could be degraded in vitro. The increase of protein in supernatant was increased in time dependent manner (Fig. 1M and N). Both PRFs were completely degraded in 180 min.

### cPDLs showed mesenchymal cell characteristics

The primary cell cultures were established from canine periodontal ligament tissues using explantation technique. The cPDLs were able to migrate out from the tissues after incubation (Fig. 2A). cPDLs exhibited a condense homogenous fibroblast-like morphology with spindle-shape appearance (Fig. 2B). When cells were seeded in low concentration, the colony formation was observed, suggesting the progenitor cell characteristics (Fig. 2C). Cells in colonies exhibited homogeneous in morphology as observed after staining with Coomassie blue (Fig. 2D). The progeny of the colony forming-cells colonies showed positive immunostaining for mesenchymal stem cell markers (CD44, CD90, and CD105) but did not express a hematopoietic stem cell marker, CD45 as assessed by flow cytometry (Fig. 2E). Further, these isolated cells express *POSTN*, one of the known markers of PDL (Suppl Fig. 1). When maintaining cells in osteogenic induction medium, the positive staining of ALP enzymatic activity and alizarin red s staining was markedly observed at day 7 and 14, respectively (Fig. 2F). Correspondingly, the increase of osteogenic marker genes (*RUNX2* and *OCN*) was observed in time dependent manner (Fig. 2G). These evidence implicate the mesenchymal cell characteristics and their differentiation ability toward osteogenic lineage.

### Thai and Murrah bubaline PRF stimulated cell viability and proliferation

**Figure 3:**
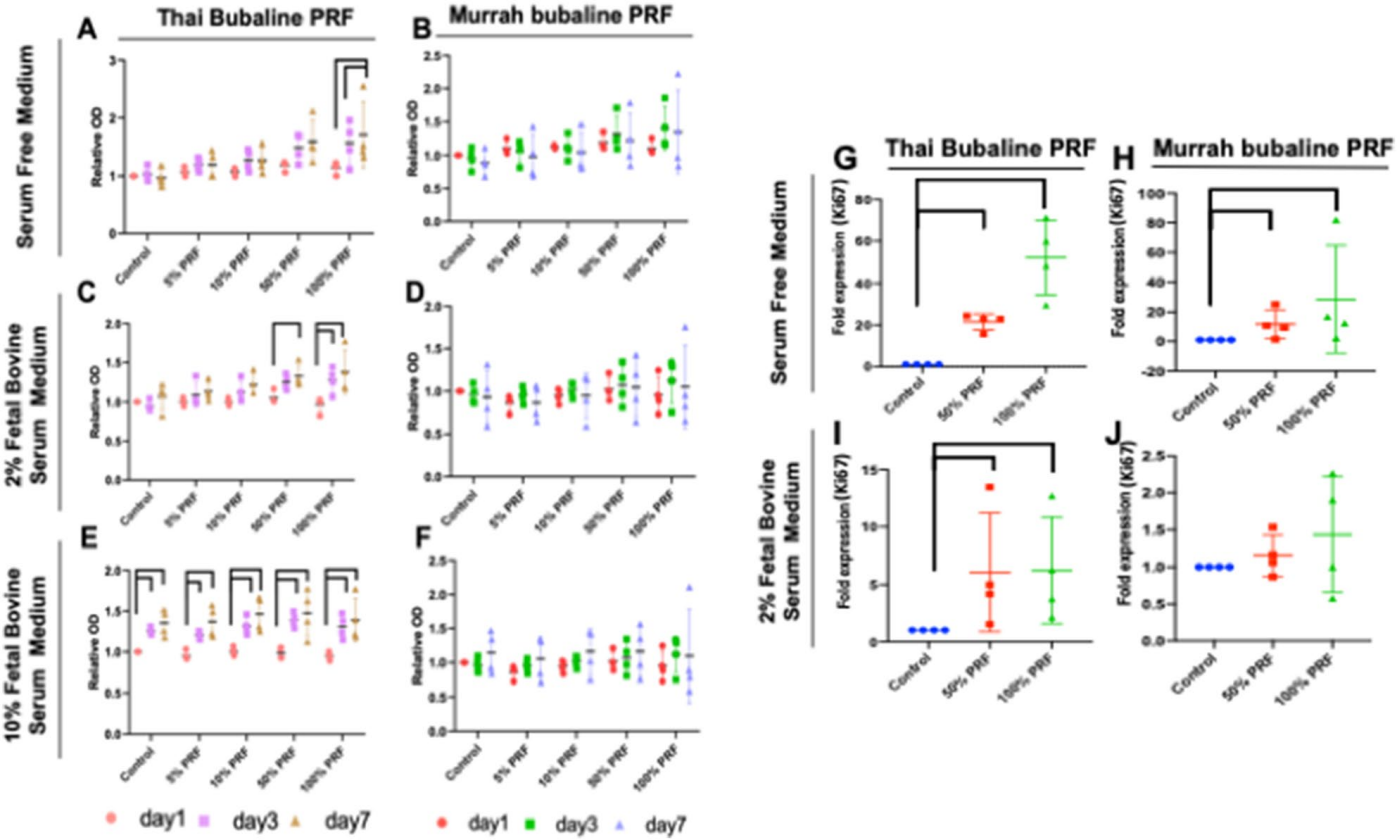
Effects of Thai and Murrah bubaline blood derived platelet rich fibrin (PRF) on cPDLs proliferation. Thai and Murrah bubaline blood derived PRF were prepared into 5%, 10%, 50%, and 100% concentration. Cells were maintained in serum free (**A** and **B**), 2% FBS (**C** and **D**), or 10% FBS (**E** and **F**) culture medium. Cell viability was examined using MTT assay at day 1, 3, and 7. cPDLs proliferation was significantly increased after treated with Thai bubaline blood derived PRF. Bars indicated the statistically significant difference. (Two-way ANOVA test with Turkey’s multiple comparisons test, *p* < 0.05). Correspondingly, Thai and Murrah bubaline blood derived PRF induced *KI67* mRNA expression in cPDLs. Thai and Murrah bubaline blood derived PRF were prepared into 50% and 100% concentration. Cells were treated with PRF for 24 h in serum free (**G** and **H**) or 2% FBS (**I** and **J**) culture medium. *KI67* mRNA levels were determined using real-time polymerase chain reaction. Bars indicated the statistically significant difference. (Mann Whitney U test, *p* < 0.05).

MTT assays were performed to examine the effects of PRF on cPDLs viability at different time points. As shown in Fig. 3, the proliferative ability of cPDLs significantly improved by increasing concentrations of fresh Thai bubaline blood derived PRF from 5% PRF to 100% PRF in the serum free and 2% FBS culture medium. The significant cell proliferation at day 3 and 7 compared with day 1 was evidenced when cPDLs were treated with 100% Thai bubaline blood derived PRF (Fig. 3A and C). At 50% Thai bubaline blood derived PRF, the significant increase of cell number was observed at day 7 compared with day 1 in 2% FBS culture medium (Fig. 3C). However, in 10% FBS culture medium, the significant increase of cell proliferation was observed in all concentration of cPDLs treated with Thai bubaline blood derived PRF (Fig. 3E). For Murrah bubaline blood derived PRF, there was an increase trend of cell number in the presence of high concentration of PRF extract. However, there was no statistically significant difference.

Further, the expression of proliferation related gene, *Ki67*, was investigated. The results showed that Thai bubaline blood derived PRF extraction at 50% and 100% significantly upregulated *Ki67* mRNA levels in cPDLs at 1 day after treatment in both serum free and 2% FBS culture medium (Fig. 3G and I). Murrah bubaline blood derived PRF extraction at 50% and 100% also promoted *Ki67* expression in serum free culture medium but not in 2% FBS culture medium (Fig. 3H and J). These results indicate that Thai bubaline blood derived PRF had a biological property to stimulate cPDLs proliferation activity.

### Bubaline PRF induced migration, spreading, and mineralization of cPDLs

**Figure 4:**
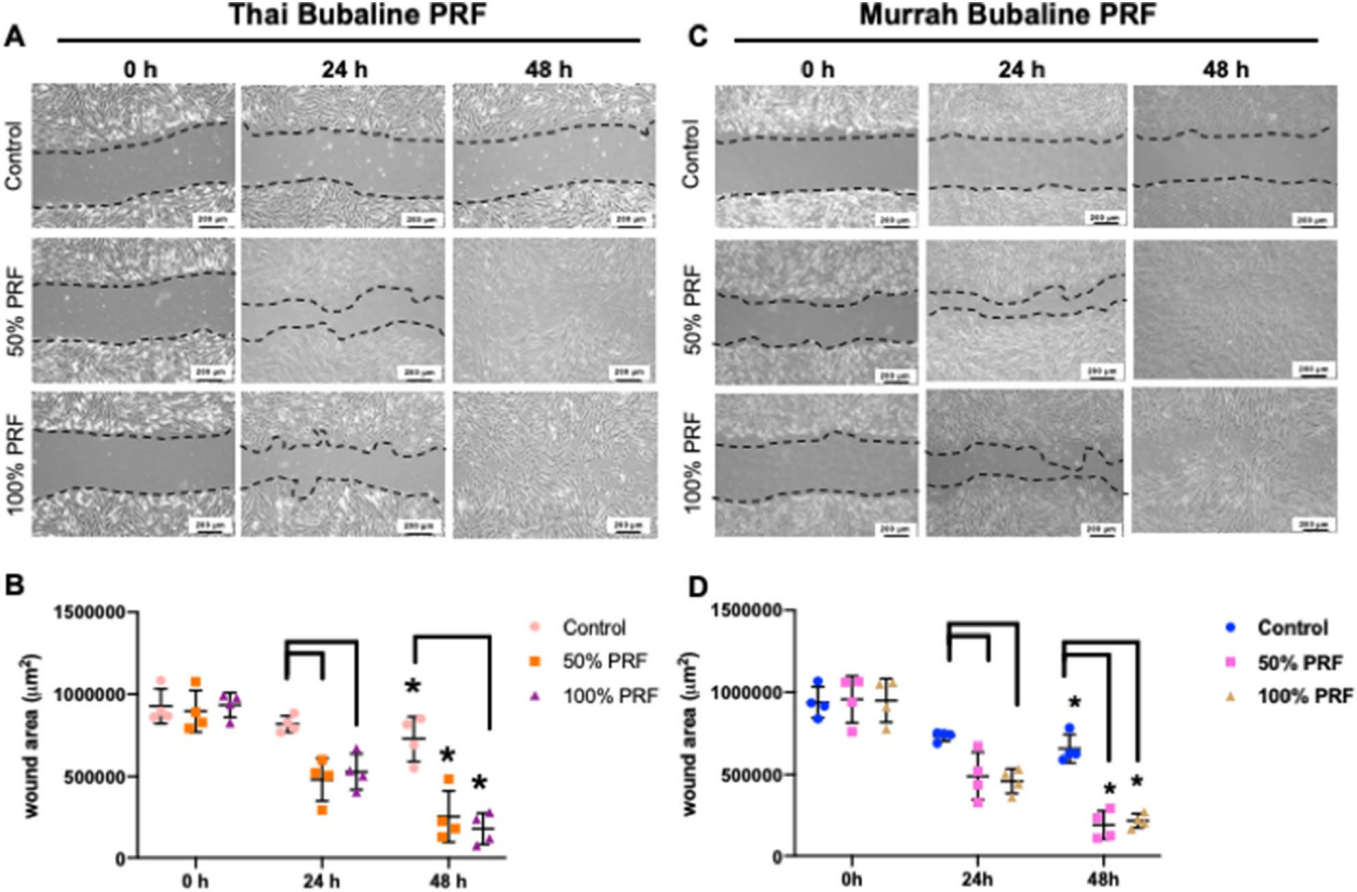
Bubaline blood derived platelet rich fibrin (PRF) induced cPDLs migration. The scratch assay was employed for cell migration study. Cells were maintained in serum free medium with the supplementation of 50% or 100% PRF extracts. The representative images of cPDLs migration were demonstrated (**A** and **C**). The scratch defect area was determined using ImageJ software. The graph demonstrated the remaining wound area (**B** and **D**). Bars indicated the statistically significant difference. Asterisks indicated the statistically significant difference compared with the same condition at 0 h. (Kruskal–Wallis test with Dunn’s multiple comparisons test, *p* < 0.05).

cPDLs were maintained in serum free culture medium in the presence of 50% and 100% PRF extracts. Cell migration was defined by closure of the scratch defect (Fig. 4). At 24 h, the marked increase of cell migration was observed in those conditions treated with PRF extracts. The scratch defects were completely filled with cPDLs at 48 h in PRF extracts treated condition.

**Figure 5:**
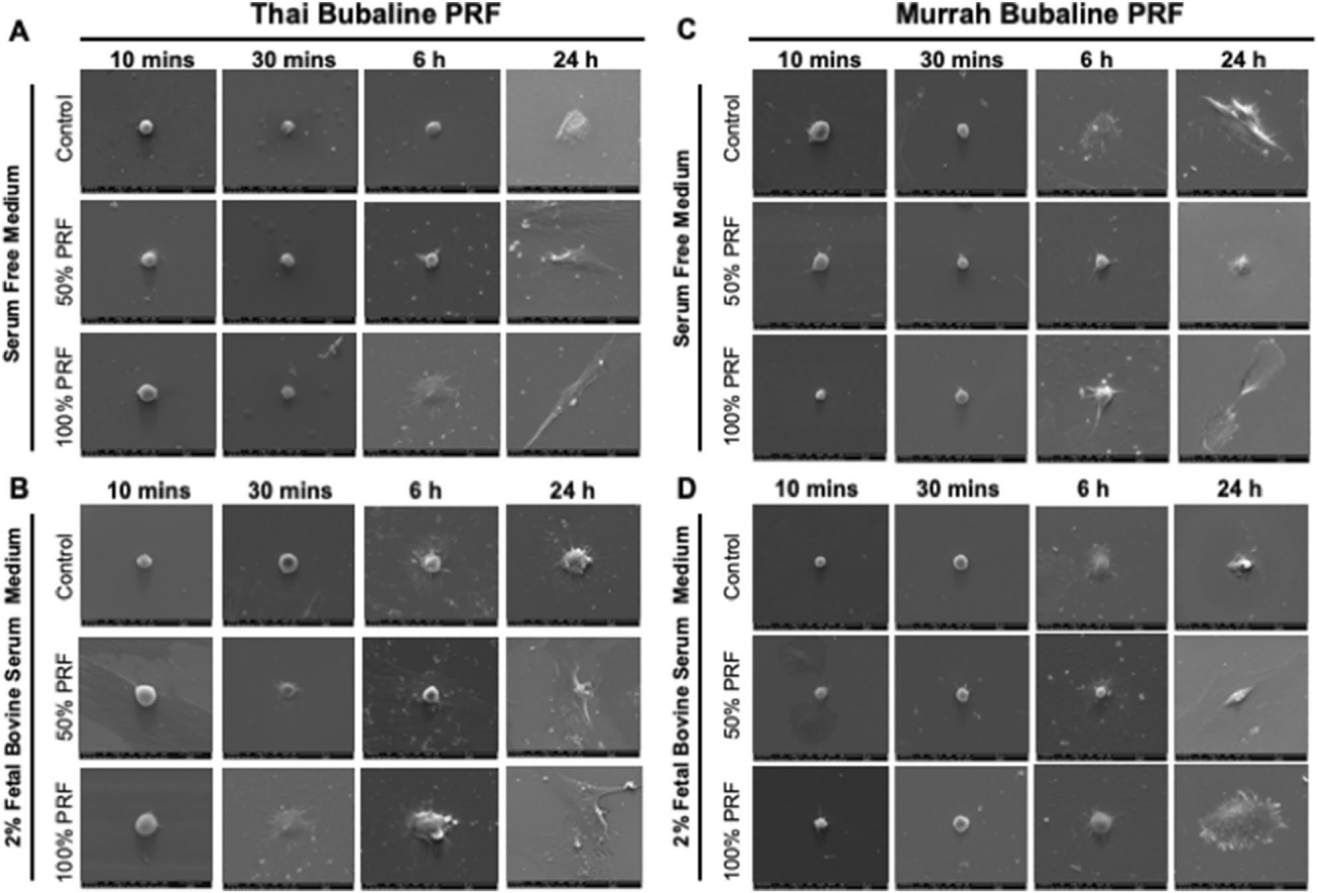
Bubaline blood derived platelet rich fibrin (PRF) induced cPDLs spreading. Cells were maintained in serum free (**A** and **C**) or 2% FBS (**B** and **D**) culture medium with the supplementation of 50% or 100% PRF extracts. Cell spreading at different time points (10 min, 30 min, 6 h and 24 h) was examined using scanning electron microscope.

Cells spreading was evaluated using SEM when cDPLs were cultured in serum free or 2% serum culture medium. SEM images of cells cultured with different concentration of Thai and Murrah bubaline blood derived PRF showed that cPDLs were attached on the surface of the glass slides within 10 min (Fig. 5). At 10 min, the round shaped cells were observed in the control condition while those cells cultured in the presence of PRF extracts exhibits some filopodia extension. At 30 min, the dramatically increase of cell spreading was observed in the condition treated with Thai bubaline blood derived extract in 2% FBS culture medium. At 6 h, cells on the control condition started to spread as the lamellopodia was observed. At 24 h, cPDLs treated with 100% PRF extracts from both Thai and Murrah bubaline blood derived PRF appeared flatten on the glass surface while those cells in the control condition still exhibited the incomplete cell spreading in morphology.

**Figure 6:**
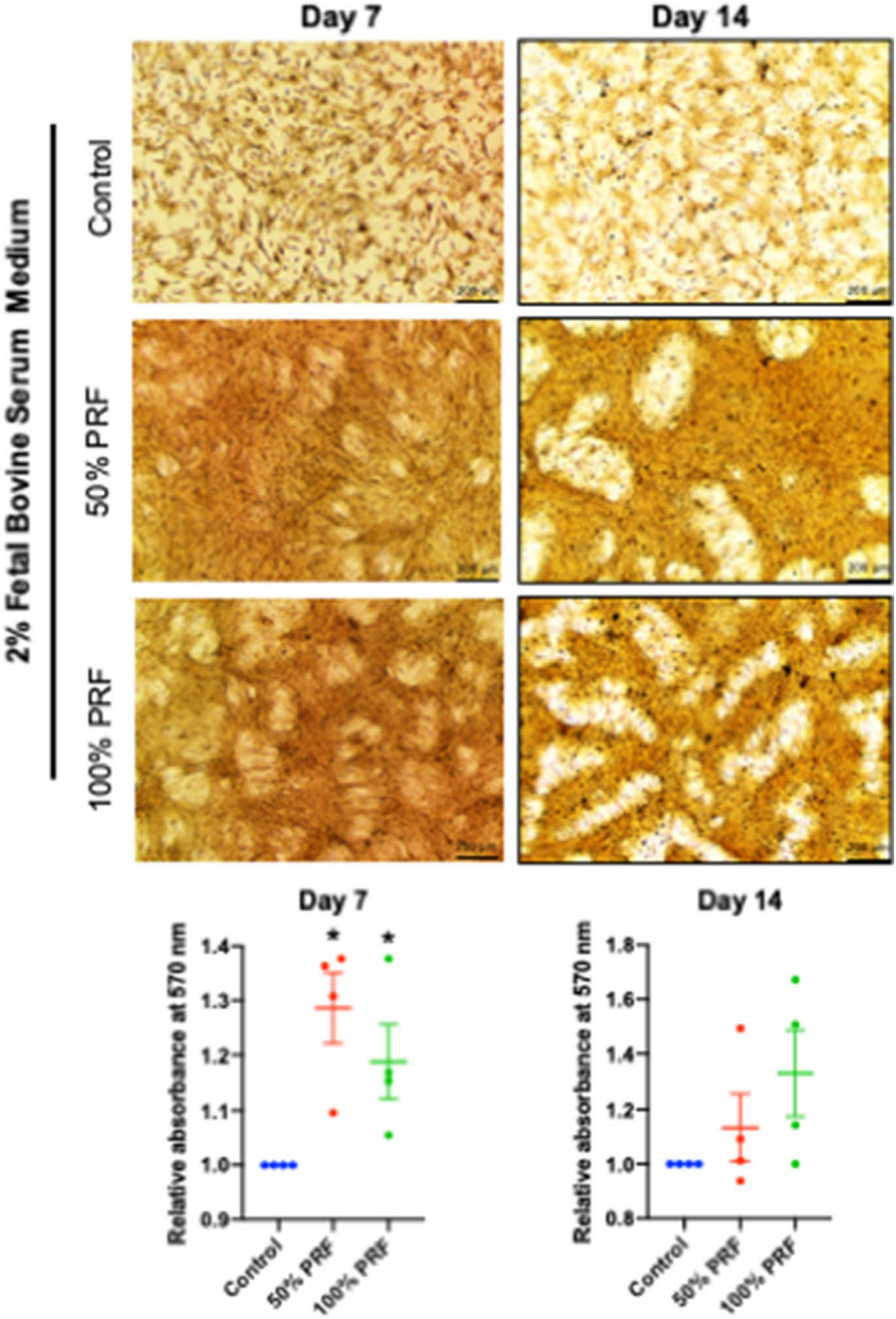
Effect of Thai bubaline blood derived platelet rich fibrin (PRF) on mineralization of cPDLs. Cells were maintained in 2% FBS culture medium supplemented with β-glycerophosphate, L-ascorbic acid, and dexamethasone for 7 and 14 day. Mineral deposition was stained using alizarin red s staining. The absorbance of solubilized stain solution was measured at 570 nm. Asterisks indicated the statistically significant different compared with the control. (Mann Whitney U test, *p* < 0.05).

To determine the effect of bubaline blood derived PRF on osteogenic differentiation of cPDLs, the mineralization assay was performed at day 7 and 14 (Fig. 6). Cells were maintained in 2% FBS culture medium supplemented with β-glycerophosphate, L-ascorbic acid, and dexamethasone in the presence of 50% or 100% Thai bubaline blood derived PRF extracts. The condition without PRF extracts supplementation was used as the control. cPDLs exposed to 50% or 100% PRF exhibited higher nodule formation and mineral deposition than the control condition at both day7 and 14. However, the significant increase of mineral deposition was observed at day 7.

The concentration of PDGF-BB was examined using ELISA. Results demonstrated that Thai bubaline blood derived PRF contained 711.38 ± 468.14 ng/ml.

### Frozen PRF also promoted cell proliferation, migration, and spreading

In order to prepare the ready to use PRF, frozen method was investigated. PRFs were frozen after collection and store in − 80 °C for 1 month. Subsequently, the PRF extraction medium was prepared in similar manner to those reported above. Thai bubaline blood derived PRF was selected in the frozen study as it was shown to contain better biological properties than Murrah bubaline blood derived PRF.

**Figure 7:**
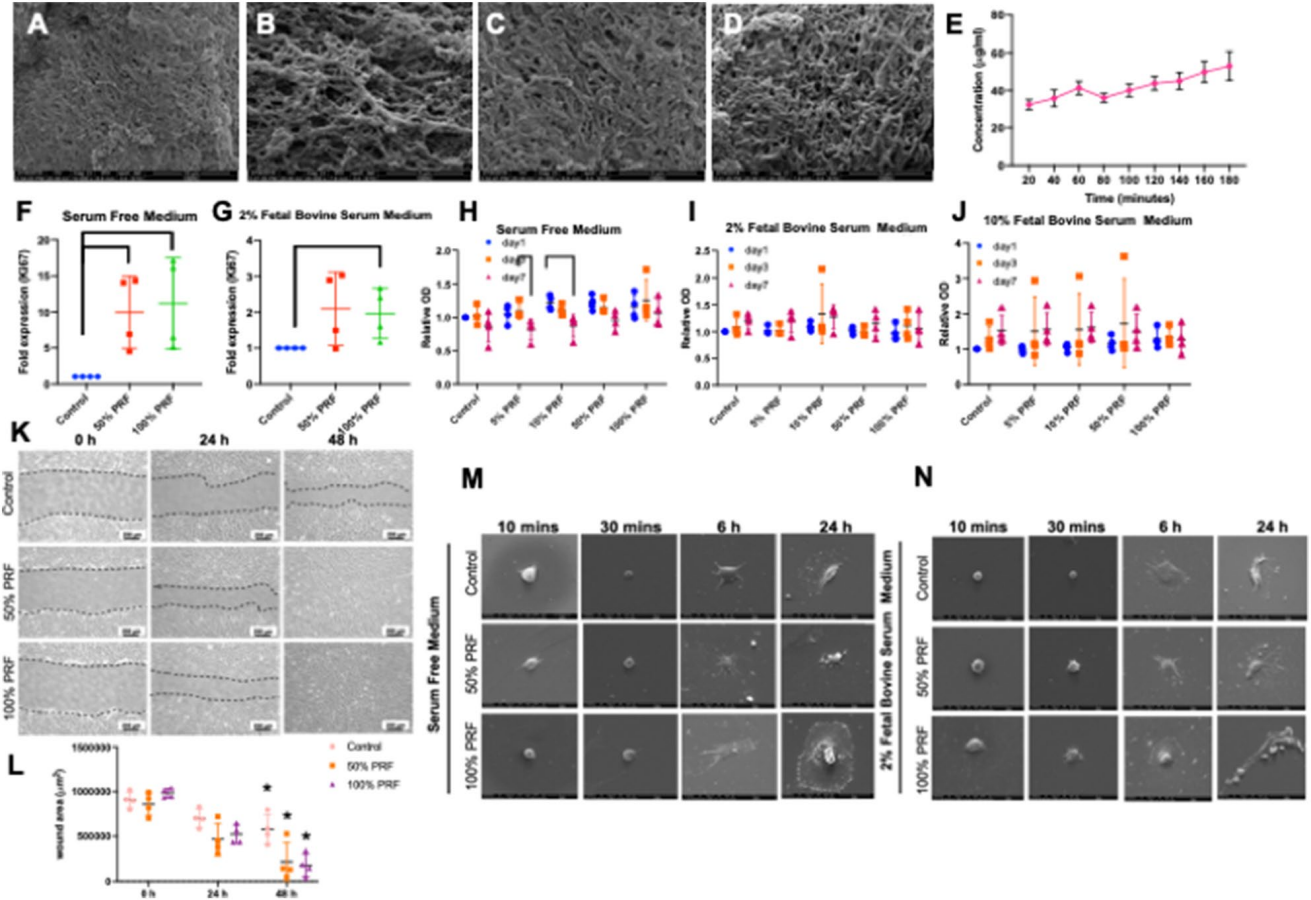
Frozen Thai bubaline blood derived platelet rich fibrin (PRF) also promoted cell proliferation, migration, and spreading. Ultrastructure of frozen Thai bubaline blood derived PRF was examined using scanning electron microscope (**A**–**D**). The in vitro degradation was determined using BCA assay (**E**). *KI67* mRNA expression was evaluated using real-time PCR (**F**–**G**). Cell viability at day 1, 3 and 7 was determined using MTT assay (**H–J**). Bars indicated the statistically significant difference. (Mann Whitney U test, *p* < 0.05). The representative images of cPDLs migration were demonstrated (**K**). The graph demonstrated the scratch defect area determining by ImageJ software (**L**). Asterisks indicated the statistically significant difference compared with the same condition at 0 h (Kruskal–Wallis test with Dunn’s multiple comparisons test, *p* < 0.05). Representative micrographs demonstrated cell morphology at different time point upon exposed to different concentration of frozen Thai bubaline blood derived PRF (**M** and **N**).

Frozen Thai bubaline blood derived PRF revealed rough and coarsen surface (Fig. 7A–D). The fibrin network showed more compact with smaller pores than those of the freshly prepared PRF. Cells entrapment within meshwork was not clearly observed. These frozen PRF could be degraded in vitro in the presence of trypsin (Fig. 7E). The degradation was time dependent manner. The frozen PRF extracts were able to induce the mRNA expression of *Ki67* at day 1 in both serum free and 2% FBS culture condition (Fig. 7F and G). However, the frozen PRF extracts failed to stimulate cell proliferation in all culture condition (Fig. 7H–J). For cell migration assay, frozen PRF extracts were able to enhance cell migration in scratch defects at 24 and 48 h compared with the control under serum free culture condition (Fig. 7K and L). No defect area was observed at 48 h in those condition treated with the frozen PRF extracts. cPDLs that were exposed to frozen PRF extracts exhibited more flatten and extension than those in the control condition at 6 and 24 h in both serum free and 2% FBS culture medium (Fig. 7M and N), indicating the facilitating cell spreading in frozen PRF treated condition.

## Discussion

Many studies have demonstrated that the subpopulation of cells isolated from periodontal ligament contains stem cell characteristics including self-renewal, mesenchymal stem cell surface marker expression, clonogenic capacity, and multipotent differentiation capability^18,19^. The characteristics of cPDLs are similar to those of human. They exhibited spindle-shaped fibroblast-like morphology, expressed mesenchymal stem cell surface markers, and were able to differentiate into osteogenic lineage^20^.

PRF is considered as a therapeutic tool for periodontal wound healing and regeneration^21–23^. It had a biocompatible, and biodegradable properties^24^. A systematic review reported that PRF promotes various biological processes in numerous cell types^25^. PRF positively influences proliferation, migration, adhesion, and osteogenic differentiation but negatively controls inflammation and osteoclastogenesis^25^. Autologous platelet rich fibrin was chosen to improve periodontal regeneration^26,27^. The study in dogs demonstrated that the combination of autologous PRF with open flap debridement improved periodontal pocket depth and cemento-enamel-junction-alveolar bone levels/root length ratio^15^. In addition, the inflammatory score was decreased in open flap debridement with PRF application compared with the control^15^, implying the beneficial utilization of PRF in periodontal regenerative treatment.

However, in clinical situation, the on-shelf, ready to use materials is preferable. Therefore, the xenogenous PRF would be useful in this requirement. Bubaline blood donation is proposed as a candidate source of this study because of its highest fibrinogen^16^. Hence, we determined in the present study on the biological response of cPDLs to bubaline blood derived PRF in vitro*.* It has been reported that bubaline fibrin glue application in pig skin grafts resulted in the higher new vessel formation and graft remaining than the control^28^. Interestingly, the xenogeneous application of bubaline fibrin glue in pig model did not exhibit the inflammatory responses^28^. In fact, the bubaline fibrin glue could reduce the histological inflammatory intensity^28^. Hence, the bubaline blood derived PRF could be a candidate source for xenogeneic materials for periodontal regeneration in dogs.

The present study utilized PDL cells isolated from dogs as cellular model. PDL samples were collection from the middle-third of the root. The cervical-third and apical-third area (upper and lower area from the red dot lines in Supply Fig. 1) have a chance to get the remaining tissues from gingiva and cellular cementum, respectively. Hence, the isolated PDL cells from middle-third of the root could prevent the compromised behaviors of other contaminated cell types. The tissue collection from specific area could minimize the heterogenicity of cell population. PDL cells highly expressed periostin and sclerostin^29^. These cells exhibited better osteogenic differentiation ability compared with gingival fibroblasts^30^. In the present study, we also confirmed that the isolated canine PDL cells exhibited PDL cell characteristics. In Supply Fig. 1, we demonstrated that canine PDL cells significantly expressed higher *POSTN* and *SOST* mRNA expression than canine gingival fibroblasts as determined using real-time polymerase chain reaction. Further, after maintaining cells in osteogenic induction medium, canine PDL cells exhibited markedly higher mineral deposition compared with canine gingival fibroblasts at day 14. Taken together, we confirmed that the isolated cells contained PDL characteristics.

The present study demonstrated that the fresh PRF preparation from Thai bubaline blood stimulated cell proliferation better than those prepared from Murrah bubaline blood, despite that both Thai and Murrah PRF stimulated *KI67* mRNA expression by cPDLs in serum free condition medium. Further, cell spreading was facilitated by Thai bubaline blood derived PRF compared with those derived from Murrah. We hypothesize that these different effects of Thai and Murrah bubaline PRF on cPDLs are from the distinct biological components in prepared PRF. All PRF collection, preparation, and extraction were performed with the same parameters hence the effect of erroneous extraction technique on different cell response is unlikely. Besides, there are several reports supporting the concept of different biological components of different bubaline species. Report showed that the swamp and Murrah buffaloes were categorized in different clades, indicating phylogenetic divergence^31^. Another study illustrated the blood chemistry difference between Thai swamp buffaloes and Mehsana riverine buffaloes^32^. These data indicate that the different responses of cPDLs to Thai and Murrah bubaline blood derived PRF might be due to the different components or concentration of PRF from Thai and Murrah buffaloes. Previous study in canine blood derived PRF illustrated that PRF contained TGF-β1 and VEGF-A protein at 170.50 ± 15.24 and 88.08 ± 10.32 mg/ml, respectively^15^. However, there is no study regarding the growth factor concentration in bubaline PRF, due to the limitation of antibody against bubaline protein.

In routine clinical practice, the preparation of PRF is easy to handle with single centrifugation and cost effective. Scanning electron microscopy of bubaline PRF show a strongly fiber dimension with numerous platelets and white blood cells entrapment. Moreover, the growth factors such as TGF-β1, VEGF, IGF, PDGF-AB, and thrombospondin were reported in the previous study^10^. Our data demonstrated that bubaline blood derived PRF contained PDGF-BB and had no cytotoxicity and significantly improved cell proliferation in cPDLs. Correspondingly, human blood derived PRF exhibited cytocompatibility toward human dental pulp stem cells and periodontal ligament stem cells in vitro^33^. Further, autologous platelet rich fibrin could recover cell viability of human periodontal ligament in the extracted teeth and kept dry for 2 h^34^. It is hypothesized that platelets and growth factors trapped within strong fibrin meshwork could gradually release growth factors and diffuse into the culture medium, subsequently influencing cell response.

Ki-67 was chosen to detect active cell proliferation as it functions in both interphase and mitotic phase^35^. Our results showed the significantly upregulation of *KI67* mRNA levels in cPDLs exposed to bubaline blood derived PRF in serum free and 2% FBS culture medium. It should be noted that the protein expression of Ki67 was not performed in the present study. Protein is the main molecule that further functions to regulate cell responses. The increase of mRNA expression may not correlate with protein expression. Hence, the upregulation of KI67 mRNA levels in PRF treated conditions may not directly influence cPDLs proliferation. Further study evaluating the mechanism(s) by which PRF induced cPDLs proliferation is indeed required. Another study reported that human periodontal ligament cells treated with platelet derived fractions had the significant higher of *KI67* expressing cells than those treated with FBS^36^. Moreover, platelet rich plasma treated C2C12 cells led to the increase of Ki67 positive cells compared to the FBS treated condition^37^. Hence, the use of platelet derived components is beneficial in term of proliferation stimulation as these products may contain higher concentration of growth factors than those in normal serum. One of the potential factors is PDGF. PDGF stimulates cell proliferation and migration via the ERK signaling pathway^38^.

Effect of PRF on cell migration has been reported as cell type dependent. In this regard, murine blood derived PRF attenuated regulatory T cell migration in transwell migration assay via the regulation of CCL2 expression and soluble CD40 ligand release^39^. Human blood derived PRF promoted human osteoblast migration better human blood derived PRP^40^. However, there was no significant effect of human PRF and PRP on human gingival fibroblast cell migration at 72 h^40^. Further, PRF has been shown to enhance endothelial cell migration^41^. Together with the present study, we reported the positive influence of bubaline blood derived PRF on cPDLs migration. These phenomenon could facilitate migration of various cell types into the defects and subsequently promote periodontal tissue healing.

Rabbit leukocyte- and PRF induced osteogenic differentiation in rabbit periodontal ligament cells^42^. In this regard, rabbit blood derived PRF significantly enhanced alkaline phosphatase enzymatic activity and osteogenic marker gene expression^42^. Correspondingly, human blood derived PRF significantly promoted alkaline phosphatase enzymatic activity and mineralization by human periodontal ligament cells^43^. Our present study demonstrated that Thai bubaline blood derived PRF could also enhance mineral deposition by cPDLs. Although, the molecular mechanism(s) is yet unclear. We hypothesize that several growth factor entrapped in PRF might responsible in this manner since it has been shown that rabbit blood derived PRF induced osteogenic marker gene expression was attenuated with anti-bFGF, anti-BMP2, and anti-TGF-β1 supplementation^42^, suggesting the involvement of these growth factors in PRF induced osteogenic differentiation processes.

Considering the ready-to-use materials, frozen PRF has been proposed as the alternative approach. Previous report illustrated that canine blood derived PRF stored in -20 °C for 1 and 2 weeks had a significant lower growth factor release compared to the freshly prepared PRF^44^. These data indicate the preferable utilization of freshly prepared PRF over the use of frozen. The biological effects of the frozen Thai bubaline blood derived PRF was investigated in our present study. Thai bubaline blood derived PRFs were stored in − 80 °C for 1 month. Results showed that frozen PRF exhibited the compromised biological activity compared to the fresh preparation. In this regard, the mesh-like structure was denser and the ability to promote cPDLs proliferation was decreased. However, the effects on cell migration and spreading of frozen PRF was similar to those of freshly prepared PRF. Hence, the frozen PRF demonstrated the compromised biological activity in vitro. The in vivo study should be performed to evaluate the possible use of frozen PRF in clinical application.

The detailed mechanism by which bubaline blood derived PRF modulates cPDLs response is not yet clearly identified. One of the limitation is the lack of specific antibody for bubaline specimens. Hence, the levels of active ingredient in bubaline blood derived PRFs can not be fully discovered. The response of different mesenchymal stem cells for periodontal regeneration is required for further investigation to understanding the mechanism of bubaline blood derived PRF. The optimal dosage or concentration of bubaline blood derived PRF would be further described.

The present study is the first to demonstrate the biological response of cPDLs on Thai and Murrah bubaline blood derived PRF in vitro*.* Freshly prepared Thai bubaline blood derived PRF exhibited the superior biological properties by stimulating cell proliferation, migration, spreading and osteogenic differentiation compared with frozen Thai or fresh Murrah bubaline blood derived PRF. These evidences could have significant clinical implications. However, the further in vivo studies are required to prove the effectiveness in animal models.

## Materials and methods

### PRF Preparation

All animal studies were approved by Chulalongkorn University Animal Care and Use Committee (Animal Use Protocol #1,931,035) and all methods were carried out in accordance with relevant guidelines and regulations. The study was carried out in compliance with the ARRIVE guidelines. Fresh blood samples were collected from Thai and Murrah buffalo (10 mL) and transferred to glass tube without anticoagulants. Thai and Murrah buffaloes were recruited in the present study due to the abundant availability in Thailand. Animals were free from zoonotic diseases such as Brucellosis, Leptospirosis, Tuberculosis, Bovine viral diarrhea, and Trypanosomiasis. The samples were centrifuged immediately by a laboratory centrifuge (Hettich Zentrifugen EBA 20, Germany) at 3000 rpm for 10 min^45^. PRF clot was collected from the middle layer of acellular plasma and red blood cells. The clot was separated using scissors. The clots were then compressed with sterile gauze to drive out the fluid. PRF membranes were cut into size 1 × 1x1 cm and stored in 5 ml serum free media overnight. The original concentration of PRF extraction medium was defined as 100% PRF and various concentration were prepared by dilution with serum free media. The concentration of 100% PRF, 50% PRF, 10% PRF, and 5% PRF were used in this study. For frozen PRF, samples were frozen in − 80 °C for 1 month and then the extracted medium was prepared in the similar manner described above.

### Scanning electron microscope analysis

The specimens were fixed with a 3% glutaraldehyde buffer at 4 °C for 1 h and subsequently passed through the serial concentration of ethanol for dehydration and processed for critical point dried step. The ultrastructural analysis was examined under scanning electron microscope (Quanta 250, FEI, Hillsboro, OR, USA).

### Degradation assay

PRF clot size 6 × 6 × 1 mm was rinsed in sterile PBS, soaked into 0.25% trypsin EDTA and incubated at 37 °C. The supernatant (50 µl) was aliquoted every 20 min. The protein concentration in the solution was examined using Pierce BCA protein assay kit according manufacturer’s protocol (Thermo Scientific, IL, USA).

### Cell culture

Primary cPDLs were isolated from freshly extracted of healthy permanent canine (second, third) premolar, 1–3 years old, male or female, mesocephalic breeds. The isolation procedures were performed using cell explant technique as previously described^30,46^ with minor modifications. Briefly, teeth were rinsed with phosphate buffer solution (PBS). PDL was gently scraped from the middle third of the root surface to prevent contamination from gingiva and cellular cementum (Suppl Fig. 1). Tissues were placed into 35 mm culture dishes containing Dulbecco’s Modified Eagle’s Medium (DMEM, Gibco, USA) supplemented with 10% fetal bovine serum (FBS, Gibco, USA), 100 μ/mL of penicillin, 2 mM L-glutamine, and 100 mg/mL of streptomycin and incubated at 37 °C in 5% carbon dioxide. The medium was changed every 2 days. After reaching confluence, cells were sub-cultured using trypsin/EDTA solution at 1:3 ratio. The cells from the second to the seventh passage were used in the following experiments.

### Flow cytometry analysis

cPDLs (1.5 × 10^7^ cells) were washed with 1% PBS and then incubated with antibodies. The antibodies were FITC conjugated mouse anti-human CD44 (BD Bioscience Pharmingen, USA), PerCP conjugated mouse anti-human CD45 (Immuno Tools, Germany), APC conjugated mouse anti-human CD90 (Immuno Tools, Germany), and PE conjugated mouse anti-human CD105 (Immuno Tools, Germany). Cells were analyzed using FACsCalibur using the CellQuest software (BD CellQuest Pro 6.1, BD Bioscience, USA).

### Colony forming assay

Cells were seeded into a 60-mm culture dishes at density of 500 cells/dish and maintained in normal growth medium. The culture medium was changed every 48 h. At 14 days, cells were fixed with 10% formalin buffer solution for 10 min and stained with Coomassie Blue (Sigma, USA) for 30 min.

### Osteogenic differentiation

Cells were seeded into 24-wells- plate at density of 5.0 × 10^4^ cells/well and maintained in normal growth medium for 24 h. Subsequently, the culture medium was replaced with osteogenic induction medium (normal culture medium supplemented with 50 µg/ml ascorbic acid, 5 mM ß-glycerophosphate and 100 nM dexamethasone). Medium was changed every 48 h.

### ALP and Mineralization assay

At day 7, cells were washed with 1% PBS, fixed with 4% formalin for 10 min, and stained with 250 µl ALP substrate solution, BCIP/NBT tablets (Roche, USA) and incubated at room temperature for 30 min. At day 14 after osteogenic induction, cells were fixed with cold methanol for 10 min. Mineral deposition was examined using alizarin red s staining. The stained mineral crystals were solubilized in 10% cetylpyridinium chloride monohydrate (Sigma-Aldrich, MO, USA) solution. The absorbance of destained solution was measured at 570 nm.

### Cell viability assay

Cell viability was evaluated using MTT assay. Cells at the density of 3.125 × 10^3^ cells/well in 96-wells-plate were incubated with different concentrations of bubaline blood-derived PRF extracted medium (5%, 10%, 50%, and 100% PRF). At day 1, 3, and 7, cells were exposed 1 mg/ml of MTT (tetrazolium-based colorimetric) solution (USB Corporation, Austria) for 15 min at 37 °C to allow formazan crystal precipitation. The formazan crystals were dissolved with dimethyl sulfoxide-glycine buffer and glycine buffer. The spectrophotometric absorbance of each sample was recorded at 570 nm (Biotek Instruments, USA).

#### Cell migration assay

Cells were seeded at a density of 2 × 10^6^ cells into 35-mm culture dishes and allowed to form a 100% confluent monolayer. Cells were starved with serum free media for 4 h and then scraped a horizontal cross of consistent width by micropipette tip in the center of the plate to create an in vitro wound. Cells were washed with PBS and replaced with serum free medium containing 50% or 100% PRF. Cells in the scraped wound area were observed under a phase-contrast microscope at × 10 magnification and measured the wound area. Photographed of the migration of the cells into the scratch area were taken at 0, 24, and 48 h. The wound area was analyzed using the ImageJ software version 1.44 for Windows program (Version 1.44, U. S. National Institutes of Health, Bethesda, Maryland, USA).

#### Cell morphology assay

Cells were seeded at density of 1.25 × 10^4^ cells into 24-wells-plate. After 24 h incubation, cells were treated with 50% or 100% PRF in either serum free medium or 2% fetal bovine serum medium. At 10 min, 30 min, 6 h, and 24 h, the sample were then fixed and processed for scanning electron microscope using aforementioned protocol.

#### Enzyme-linked immunosorbent assay

The PRF membranes were cut into 6 × 6 mm pieces with a 6-mm biopsy punch and homogenized using homogenizing pestles. After centrifuging at 3000 rpm for 10 min at 4 °C, the supernatant was collected and the concentration of PDGF-BB was measured using ELISA kits according to the manufacturer’s protocol (Mybiosource, San Diego, CA, USA): PDGF-BB (Cat. No. MBS0033120).

#### Real-time polymerase chain reaction (PCR)

Total cellular RNA was isolated using trizol reagent (RiboEx, Geneall Biotechnology, Korea). The extracted RNA was processed to cDNA using a reverse transcriptase reaction (Promega, Madison, WI, USA). PCR was performed using a CFX Connect Real-Time PCR detection system (Biorad, USA) with FastStart Essential DNA Green Master (Roche Applied Science). The reaction condition was denaturing at 95 °C for 5 min followed by forty amplification cycles. The amplification cycle condition consisted of denaturing at 95 °C for 10 s, annealing at 60 °C for 10 s, and extension at 72 °C for 25 s, a final extension step at 72 °C for 20 min. Product specificity was confirmed by post-amplification melting curve analysis. The final expression levels were normalized to *b-ACTIN* expression levels. The oligonucleotide sequences were as followed; *ACTB* (XM_005621019.3) Forward: 5’-GCAAGGACCTCTATGCCAACA-3’, Reverse: 5’- GAAGCATTTGCGGTGGACG-3’, size 257 bp; *KI67* (XM 014,108,788.1) Forward: 5’- GTGCAACTAAAGCACGGAGA -3’, Reverse: 5’- GAGATTCCTGTTTGCGTTTTCG -3’, size 124 bp; *OCN* (XM_014115322.2) Forward: 5’- TCACAGACCCAGACAGAACCG -3’, Reverse: 5’- AGCCCAGAGTCCAGGTAGCG -3’, size 207 bp; *RUNX2* (XM_022425793.1) Forward: 5’- TCCAGACCAGCAGCACTCCATA -3’, Reverse: 5’- TTCCATCAGCGTCAACACCATC -3’, size 186 bp.

#### Statistical analyses

Ten animals from each species were recruited for blood collection and PRF preparation. Physical characterization of PRF was performed from biological triplicate. PRF extraction media from all donors were pooled for cell study. For cell study, the experiments were performed in biological quadruplicate. Data are expressed as mean ± S.D. Difference among groups was determined using Kruskal Wallis test followed by a pairwise comparison. For two groups comparison, Mann Whitney U test was employed. Difference of cell proliferation at different time points in various PRF concentration was evaluated using two-way ANOVA test followed by Tukey’s post hoc test for multiple comparisons. The statistical analysis was performed using Prism8 version 8.4.0 for Windows program (GraphPad Software version 8.4.0, CA, USA). The statistical significance was considered at *P* < 0.05.

## Supplementary Information

Supplementary Information.
