# Supplementary material for: Responses of canine periodontal ligament cells to bubaline blood derived platelet rich fibrin in vitro
Source: Sci Rep. 2021 Jun 1;11:11409. doi: 10.1038/s41598-021-90906-z (PMC8169705; doi:10.1038/s41598-021-90906-z)
Supplement: Supplementary file 1 — Supplementary Information. [file 41598_2021_90906_MOESM1_ESM.docx]

**Responses of canine periodontal ligament cells to bubaline blood derived platelet rich fibrin *in vitro***

Poranee Banyatworakul^1^, Thanaphum Osathanon^2,3,*^, Sujin Chumprasert^4^, Prasit Pavasant^3^, Nopadon Pirarat^1,5,*^

^1^Department of Pathology, Faculty of Veterinary Science, Chulalongkorn University, Bangkok, 10330 Thailand

^2^Dental Stem Cell Biology Research Unit, Faculty of Dentistry, Chulalongkorn University, Bangkok, 10330 Thailand

^3^Center of Excellence for Regenerative Dentistry and Department of Anatomy, Faculty of Dentistry, Chulalongkorn University, Bangkok, 10330 Thailand

^4^Oral Biology Research Center, Faculty of Dentistry, Chulalongkorn University, Bangkok, 10330 Thailand

^5^Wildlife Exotic and Aquatic Pathology-Research Unit, Department of Pathology, Faculty of Veterinary Science, Chulalongkorn University, Bangkok, 10330 Thailand

***Corresponding author:**

Nopadon Pirarat, D.V.M., Ph.D.

Wildlife Exotic and Aquatic Pathology-Research Unit, Department of Pathology, Faculty of Veterinary Science, Chulalongkorn University, Bangkok, 10330 Thailand

E-mail: [nopadonpirarat@gmail.com](mailto:nopadonpirarat@gmail.com)

Thanaphum Osathanon, D.D.S., Ph.D.

Faculty of Dentistry, Chulalongkorn University, Bangkok, 10330 Thailand

Email: [thanaphum.o@chula.ac.th](mailto:thanaphum.o@chula.ac.th)


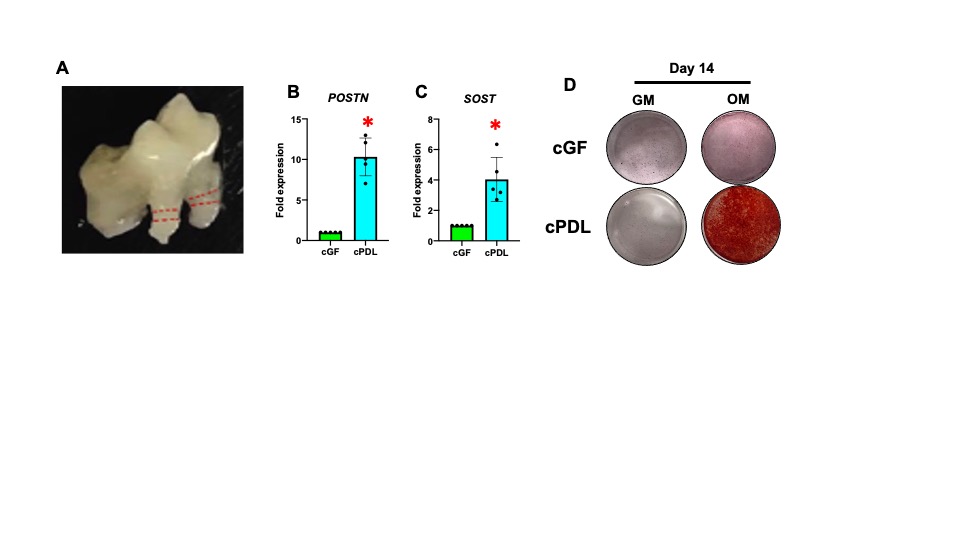


**Supplementary Figure 1** (A) Representative images demonstrated the location of root that PDL cells were isolated (between red dot lines). (B) *POSTN* and (C) *SOST* mRNA expression was determined using real-time polymerase chain reaction. *ACTB* (XM_005621019.3) Forward: 5’-GCAAGGACCTCTATGCCAACA-3’, Reverse: 5’- GAAGCATTTGCGGTGGACG-3’, size 257 bp; *POSTN* (XM_003433308.4) Forward: 5’- TGTTGCCCTGGTTATATGAG-3’, Reverse: 5’- ACTCGGTGCAAAGTAAGTGA-3’, size 180 bp; *SOST* (NM_025237.3) Forward: 5’-ACTTCAGAGGAGGCAGAAATGG-3’,

Reverse: 5’-CAAGGGGGAATCTTATCCAACTTTC-3’. (D) Cells were cultured in osteogenic induction medium for 14 days and mineral deposition was examined using alizarin red s staining. cGF: canine gingival fibroblast cells; cPDL: canine periodontal ligament cells; GM: growth medium; OM: osteogenic induction medium. Asterisks indicated the statistically significant difference (p<0.05).
